# Supplementary material for: Comparison of Conventional and Radiomic Features between 18F-FBPA PET/CT and PET/MR
Source: Biomolecules. 2021 Nov 9;11(11):1659. doi: 10.3390/biom11111659 (PMC8615400; doi:10.3390/biom11111659)
Supplement: Supplementary file 1 [file biomolecules-11-01659-s001.zip › biomolecules-1420074-supplementary.pdf]

SUPPLEMENTAL MATERIALS

for

Comparison of Routine and Radiomic Features between ^{18}F-FBPA PET/CT and PET/MR Imaging

by

Chien-Yi Liao, Jun-Hsuang Jen, Yi-Wei Chen, Chien-Ying Li, Ling-Wei Wang, Ren-Shyan Liu, Wen-Sheng Huang*, and Chia-Feng Lu*

Table S1 The formulae for the calculation of primary radiomic features.

**Intensity-based features (first order statistics)**<br>**X** denotes the intensity vector with *N* voxels of the tumor ROIs; $\overline{\mathrm{X}}$, the mean of **X**; **P**, the first-order histogram with N_l discrete intensity levels.			
**Feature**	**Formula**	**Feature**	**Formula**
1. Energy	$\sum_{i=1}^{N}\mathbf{X}(i)^2$	2. Entropy	$\sum_{i=1}^{N_l}\mathbf{P}(i)log_2\mathbf{P}(i)$
3. Kurtosis	$\frac{\frac{1}{N}\sum_{i=1}^{N}(\mathbf{X}(i)-\bar{X})^4}{\left(\sqrt{\frac{1}{N}\sum_{i=1}^{N}(\mathbf{X}(i)-\bar{X})^2}\right)^2}-3$	4. Maximum	$\max(\mathbf{X})$
5. Mean	$\frac{1}{N}\sum_{i=1}^{N}\mathbf{X}(i)$	6. Mean absolute deviation	$\frac{1}{N}\sum_{i=1}^{N}\mathrm{abs}(\mathbf{X}(i)-\bar{X})$
7. Median	$\mathrm{median}(\mathbf{X})$	8. First quartile	Value that splits off the lowest 25% of data from the highest 75%
9. Third quartile	Value that splits off the highest 25% of data from the lowest 75%	10. Minimum	$\min(\mathbf{X})$
11. Range	$\max(\mathbf{X})-\min(\mathbf{X})$	12. Root mean square (RMS)	$\sqrt{\frac{\sum_{i=1}^{N}\mathbf{X}(i)^2}{N}}$
13. Skewness	$\frac{\frac{1}{N}\sum_{i=1}^{N}(\mathbf{X}(i)-\bar{X})^3}{\left(\sqrt{\frac{1}{N}\sum_{i=1}^{N}(\mathbf{X}(i)-\bar{X})^2}\right)^3}$	14. Standard deviation	$\sqrt{\frac{1}{N}\sum_{i=1}^{N}(\mathbf{X}(i)-\bar{X})^2}$
15. Uniformity	$\sum_{i=1}^{N_l}\mathbf{P}(i)^2$	16. Variance	$\frac{1}{N}\sum_{i=1}^{N}(\mathbf{X}(i)-\bar{X})^2$
**Shape- and Size-based features**<br>*V*, tumor volume; *A*, surface area of the volume			
17. Compactness 1	$\frac{V}{\sqrt{\pi}A^{3/2}}$	18. Compactness 2	$36\pi\frac{V^2}{A^3}$
19. Maximum 3D diameter	The largest pairwise Euclidean distance between voxels on the surface of the tumor volume.	20. Spherical disproportion	$\frac{A}{4\pi R^2}$
21. Sphericity	$\frac{\pi^{\frac{1}{3}}(6V)^{\frac{2}{3}}}{A}$	22. Surface area	$\mathrm{A}=\sum_{i=1}^{Ns}\frac{1}{2}\lvert a_ib_i \times a_ic_i\rvert$<br>*Ns*, total number of triangles covering the surface; *a*, *b*,

			and *c*, triangle vertices
23. Surface to volume ratio	$\frac{A}{V}$	24. Volume	Number of pixels in the tumor region multiplied by the voxel size

Textural features (gray-level co-occurrence matrix based features)

$\mathbf{P}(\delta,\alpha)$, co-occurrence matrix for an arbitrary distance δ and direction α; N_g , number of discrete intensity levels in the image; p_x(i), marginal row probabilities; p_y(i), marginal column probabilities; μ_x, mean of p_x; μ_y, mean of p_y; σ_x, standard deviation of p_x; σ_y, standard deviation of p_y; *HXY*, entropy of **P**; *HX*, entropy of p_x; *HY* , entropy of p_y;

$$p_{x+y}(k) = \sum_{i=1}^{N_g}\sum_{j=1}^{N_g}\mathbf{P}(i,j)\,, i+j=k, k=2,3,\dots,2N_g;$$

$$p_{x-y}(k) = \sum_{i=1}^{N_g}\sum_{j=1}^{N_g}\mathbf{P}(i,j)\,, |i-j|=k, k=0,1,\dots,N_g-1;$$

$$\mathrm{HX} = -\sum_{i=1}^{N_g} p_x(i)\log_2\big(p_x(i)\big),\ \ \mathrm{HY} = -\sum_{i=1}^{N_g} p_y(i)\log_2\left(p_y(i)\right);$$

$$\mathrm{HXY1} = -\sum_{i=1}^{N_g}\sum_{j=1}^{N_g}\mathbf{P}(i,j)\log_2(p_x(i)p_y(j))\,,\ \ \mathrm{HXY2} =$$

$$-\sum_{i=1}^{N_g}\sum_{j=1}^{N_g} p_x(i)p_y(j)\log_2(p_x(i)p_y(j))$$

25. Autocorrelation	$\sum_{i=1}^{N_g}\sum_{j=1}^{N_g} ij\mathbf{P}(i,j)$	26. Cluster Prominence	$\sum_{i=1}^{N_g}\sum_{j=1}^{N_g}[\boldsymbol{i}+\boldsymbol{j}-\boldsymbol{\mu_x}-\boldsymbol{\mu_y}]^4\mathbf{P}(i,j)$
27. Cluster Shade	$\sum_{i=1}^{N_g}\sum_{j=1}^{N_g}[\boldsymbol{i}+\boldsymbol{j}-\boldsymbol{\mu_x}-\boldsymbol{\mu_y}]^3\mathbf{P}(i,j)$	28. Cluster Tendency	$\sum_{i=1}^{N_g}\sum_{j=1}^{N_g}[\boldsymbol{i}+\boldsymbol{j}-\boldsymbol{\mu_x}-\boldsymbol{\mu_y}]^2\mathbf{P}(i,j)$
29. Contrast	$\sum_{i=1}^{N_g}\sum_{j=1}^{N_g}\lvert\boldsymbol{i}-\boldsymbol{j}\rvert^2\mathbf{P}(i,j)$	30. Correlation	$\sum_{i=1}^{N_g}\sum_{j=1}^{N_g}\frac{ij\mathbf{P}(i,j)-\mu_x(i)\mu_y(j)}{\sigma_x(i)\sigma_y(j)}$
31. Difference entropy	$\sum_{i=0}^{N_g-1} p_{x-y}(i)log_2[p_{x-y}(i)]$	32. Dissimilarity	$\sum_{i=1}^{N_g}\sum_{j=1}^{N_g}\lvert\boldsymbol{i}-\boldsymbol{j}\rvert\mathbf{P}(i,j)$
33. Energy	$\sum_{i=1}^{N_g}\sum_{j=1}^{N_g}[\mathbf{P}(i,j)]^2$	34. Entropy (*HXY*)	$-\sum_{i=1}^{N_g}\sum_{j=1}^{N_g}\mathbf{P}(i,j)\log_2(\mathbf{P}(i,j))$
35. Homogeneity 1	$\sum_{i=1}^{N_g}\sum_{j=1}^{N_g}\frac{\mathbf{P}(i,j)}{1+\lvert i-j\rvert}$	36. Homogeneity 2	$\sum_{i=1}^{N_g}\sum_{j=1}^{N_g}\frac{\mathbf{P}(i,j)}{1+\lvert i-j\rvert^2}$
37. Informational measure of correlation 1	$\frac{HXY-HXY1}{\max(HX,HY)}$	38. Informational measure of correlation 2	$\sqrt{1-e^{-2(HXY2-HXY)}}$

39. Inverse Difference Moment Normalized	$\sum_{i=1}^{N_g}\sum_{j=1}^{N_g}\frac{\mathbf{P}(i,j)}{1+\left(\frac{\lvert i-j\rvert^2}{N^2}\right)}$	40. Inverse Difference Normalized	$\sum_{i=1}^{N_g}\sum_{j=1}^{N_g}\frac{\mathbf{P}(i,j)}{1+\left(\frac{\lvert i-j\rvert}{N}\right)}$
41. Inverse variance	$\sum_{i=1}^{N_g}\sum_{j=1}^{N_g}\frac{\mathbf{P}(i,j)}{\lvert i-j\rvert^2}, i \neq j$	42. Maximum Probability	$\max(\mathbf{P}(i,j))$
43. Sum average	$\sum_{i=2}^{2N_g}[i\mathbf{P}_{x+y}(i)]$	44. Sum entropy	$-\sum_{i=2}^{2N_g}\mathbf{P}_{x+y}(i)log_2[\mathbf{P}_{x+y}(i)]$
45. Variance	$\sum_{i=1}^{N_g}\sum_{j=1}^{N_g}(i-\mu)^2\mathbf{P}(\mathbf{i},\mathbf{j})$		
**Textural features (gray-level run-length matrix based features)** $p(i,j\|\theta)$, (*i,j*)th entry in the given run-length matrix *p* for a direction ϑ; N_g, number of discrete intensity levels in the image; N_r, number of different run lengths			
46. Short Run Emphasis	$\frac{\sum_{i=1}^{N_g}\sum_{j=1}^{N_r}\left[\frac{p(i,j\|\theta)}{j^2}\right]}{\sum_{i=1}^{N_g}\sum_{j=1}^{N_r}p(i,j\|\theta)}$	47. Long Run Emphasis	$\frac{\sum_{i=1}^{N_g}\sum_{j=1}^{N_r}j^2p(i,j\|\theta)}{\sum_{i=1}^{N_g}\sum_{j=1}^{N_r}p(i,j\|\theta)}$
48. Gray Level Non-Uniformity	$\frac{\sum_{i=1}^{N_g}\left[\sum_{j=1}^{N_r}p(i,j\|\theta)\right]^2}{\sum_{i=1}^{N_g}\sum_{j=1}^{N_r}p(i,j\|\theta)}$	49. Run Length Non-Uniformity	$\frac{\sum_{j=1}^{N_r}\left[\sum_{i=1}^{N_g}p(i,j\|\theta)\right]^2}{\sum_{i=1}^{N_g}\sum_{j=1}^{N_r}p(i,j\|\theta)}$
50. Run Percentage	$\sum_{i=1}^{N_g}\sum_{j=1}^{N_r}\frac{p(i,j\|\theta)}{N_p}$	51. Low Gray Level Run Emphasis	$\frac{\sum_{i=1}^{N_g}\sum_{j=1}^{N_r}\left[\frac{p(i,j\|\theta)}{i^2}\right]}{\sum_{i=1}^{N_g}\sum_{j=1}^{N_r}p(i,j\|\theta)}$
52. High Gray Level Run Emphasis	$\frac{\sum_{i=1}^{N_g}\sum_{j=1}^{N_r}i^2p(i,j\|\theta)}{\sum_{i=1}^{N_g}\sum_{j=1}^{N_r}p(i,j\|\theta)}$	53. Short Run Low Gray Level Emphasis	$\frac{\sum_{i=1}^{N_g}\sum_{j=1}^{N_r}\left[\frac{p(i,j\|\theta)}{i^2j^2}\right]}{\sum_{i=1}^{N_g}\sum_{j=1}^{N_r}p(i,j\|\theta)}$
54. Short Run High Gray Level Emphasis	$\frac{\sum_{i=1}^{N_g}\sum_{j=1}^{N_r}\left[\frac{p(i,j\|\theta)i^2}{j^2}\right]}{\sum_{i=1}^{N_g}\sum_{j=1}^{N_r}p(i,j\|\theta)}$	55. Long Run Low Gray Level Emphasis	$\frac{\sum_{i=1}^{N_g}\sum_{j=1}^{N_r}\left[\frac{p(i,j\|\theta)j^2}{i^2}\right]}{\sum_{i=1}^{N_g}\sum_{j=1}^{N_r}p(i,j\|\theta)}$
56. Long Run High Gray Level Emphasis	$\frac{\sum_{i=1}^{N_g}\sum_{j=1}^{N_r}i^2j^2p(i,j\|\theta)}{\sum_{i=1}^{N_g}\sum_{j=1}^{N_r}p(i,j\|\theta)}$		

Table S2 The linear equations for interchanging PET/CT to PET/MR radiomics.
GLCM: gray-level co-occurrence matrix. In the column of wavelet filtering, L represents a low-pass filter, and H represents a high-pass filter. The combination of L and H letters stands for the filter type applied to the three image axes in order.

Wavelet Filtering	Feature Type	Feature Name	Linear transformation formula		Correlation
			m(slope)	b(intercept)	
None	Routine	Maximum	m=1.1481	b=-0.41739	strong
None	Routine	Mean	m=1.0459	b=-0.059898	strong
None	Routine	Minimum	m=1.0074	b=-0.022207	strong
None	Routine	T/N ratio	m=0.95149	b=0.077946	strong
None	Histogram	Root mean square	m=1.0557	b=-0.078156	strong
None	Histogram	Standard deviation	m=1.2221	b=-0.048134	strong
None	Histogram	Variance	m=1.347	b=-0.011788	strong
None	Histogram	Energy	m=1.3584	b=-5028.0672	strong
None	Geometric	Surface area	m=1.0123	b=308.6175	strong
None	Geometric	Surface to volume ratio	m=1.1079	b=-0.036636	strong
None	Geometric	Volume	m=1.1343	b=-491.7872	strong
None	GLCM	Contrast	m=1.1573	b=-2.0035	strong
None	GLCM	Correlation	m=1.1098	b=-0.082595	strong
None	GLCM	Difference entropy	m=1.0171	b=-0.061313	strong
None	Histogram	Entropy	m=1.0184	b=-0.12863	strong
None	GLCM	Dissimilarity	m=1.0525	b=-0.14794	strong
None	GLCM	Homogeneity 1	m=0.91885	b=0.033259	strong
None	GLCM	Homogeneity 2	m=0.89842	b=0.033548	strong
None	GLCM	Informational measure of correlation 1 (IMC1)	m=1.3765	b=0.069475	strong
None	GLCM	Inverse Difference Moment Normalized (IDMN)	m=1.1341	b=-0.13243	strong
None	GLCM	Inverse Difference Normalized (IDN)	m=1.0255	b=-0.023373	strong
None	GLCM	Inverse variance	m=0.91614	b=0.029593	strong
None	GLRLM	Short Run Emphasis (SRE)	m=0.90146	b=0.090528	strong
None	GLRLM	Long Run Emphasis (LRE)	m=0.88318	b=0.17176	strong
None	GLRLM	Long Run Emphasis (LRE)	m=1.0557	b=3.5191	strong
None	GLRLM	Run Length Nonuniformity (RLN)	m=1.1308	b=-60.1364	strong
None	GLRLM	Run Percentage (RP)	m=0.89441	b=0.093793	strong
None	Histogram	Maximum	m=1.1481	b=-0.41739	strong
None	GLRLM	Low Gray-Level Run Emphasis	m=0.92278	b=0.00027044	strong

		(LGLRE)			
None	GLRLM	Short Run Low Gray-Level Emphasis (SRLGLE)	m=0.92792	b=0.00019282	strong
None	Histogram	Mean	m=1.0459	b=-0.059898	strong
None	Histogram	Mean absolute deviation	m=1.214	b=-0.034528	strong
None	Histogram	First quartile	m=1.0033	b=0.013916	strong
None	Histogram	Third quartile	m=1.0564	b=-0.076298	strong
None	Histogram	Median	m=1.0204	b=-0.0080993	strong
None	Histogram	Minimum	m=1.0074	b=-0.022207	strong
None	Histogram	Range	m=1.2079	b=-0.29923	strong
HHH	GLCM	Informational measure of correlation 1 (IMC1)	m=1.4757	b=0.070997	strong
HHH	GLRLM	Long Run Emphasis (LRE)	m=1.2181	b=15.3208	strong
HHH	GLRLM	Run Length Nonuniformity (RLN)	m=1.1313	b=-122.8677	strong
HHH	Histogram	Mean	m=1.0664	b=-4.0764e-07	strong
HHL	GLCM	HHL_27_Contrast	m=1.3764	b=-3.1733	strong
HHL	GLCM	Informational measure of correlation 1 (IMC1)	m=1.5816	b=0.11411	strong
HHL	GLCM	Inverse Difference Moment Normalized (IDMN)	m=1.2847	b=-0.28258	strong
HHL	GLRLM	Long Run Emphasis (LRE)	m=1.1269	b=21.4468	strong
HHL	GLRLM	Run Length Nonuniformity (RLN)	m=1.1909	b=-191.0173	strong
HHL	Histogram	Mean	m=0.81878	b=6.8875e-05	strong
HHL	Histogram	First quartile	m=1.1725	b=0.00011033	strong
HHL	Histogram	Third quartile	m=0.76606	b=0.00052683	strong
HHL	Histogram	Median	m=0.82446	b=6.7727e-06	strong
HLH	GLCM	Informational measure of correlation 1 (IMC1)	m=1.3702	b=0.088694	strong
HLH	GLCM	Informational measure of correlation 2 (IMC2)	m=1.3146	b=-0.31444	strong
HLH	GLRLM	Long Run Emphasis (LRE)	m=1.2279	b=16.9739	strong
HLH	GLRLM	Run Length Nonuniformity (RLN)	m=1.1871	b=-221.0723	strong
HLH	Histogram	Mean	m=1.4299	b=-0.00074121	strong
HLH	Histogram	Mean absolute deviation	m=1.2671	b=-0.00072127	strong
HLH	Histogram	Third quartile	m=1.1937	b=-0.00068174	strong
HLH	Histogram	Median	m=1.03	b=-0.00015737	strong
HLL	Histogram	Root mean square	m=0.97143	b=0.0053578	strong

HLL	Histogram	Standard deviation	m=0.9552	b=0.0066208	strong
HLL	Histogram	Energy	m=1.5041	b=-3.9012	strong
HLL	GLCM	Homogeneity 1	m=0.8817	b=0.045661	strong
HLL	GLCM	Informational measure of correlation 1 (IMC1)	m=1.2812	b=0.086628	strong
HLL	GLCM	Inverse Difference Normalized (IDN)	m=1.0939	b=-0.093541	strong
HLL	GLRLM	Long Run Emphasis (LRE)	m=1.2774	b=-31.1059	strong
HLL	GLRLM	Run Length Nonuniformity (RLN)	m=1.0782	b=106.6885	strong
HLL	Histogram	Maximum	m=1.6251	b=-0.057255	strong
HLL	GLRLM	Low Gray-Level Run Emphasis (LGLRE)	m=1.5446	b=-0.0031751	strong
HLL	GLRLM	Short Run Low Gray-Level Emphasis (SRLGLE)	m=1.5081	b=-0.0026925	strong
HLL	Histogram	Mean	m=1.0295	b=0.0026863	strong
HLL	Histogram	Mean absolute deviation	m=0.95506	b=0.004957	strong
HLL	Histogram	First quartile	m=1.0371	b=0.0015943	strong
HLL	Histogram	Third quartile	m=1.0107	b=0.0051451	strong
HLL	Histogram	Median	m=1.057	b=0.004216	strong
LHH	GLCM	Informational measure of correlation 1 (IMC1)	m=1.7285	b=0.15845	strong
LHH	GLRLM	Long Run Emphasis (LRE)	m=1.3195	b=-45.6702	strong
LHH	GLRLM	Run Length Nonuniformity (RLN)	m=1.0921	b=65.0231	strong
LHH	GLRLM	Short Run Low Gray-Level Emphasis (SRLGLE)	m=0.68515	b=0.00085063	strong
LHH	Histogram	Mean	m=0.90817	b=0.0002401	strong
LHH	Histogram	Third quartile	m=0.89104	b=0.0016329	strong
LHL	Histogram	Root mean square	m=0.78272	b=0.016021	strong
LHL	Histogram	Energy	m=1.4025	b=-6.6916	strong
LHL	GLCM	Informational measure of correlation 1 (IMC1)	m=1.1895	b=0.052149	strong
LHL	GLRLM	Long Run Emphasis (LRE)	m=1.0949	b=-6.774	strong
LHL	GLRLM	Run Length Nonuniformity (RLN)	m=1.2719	b=-305.5976	strong
LHL	Histogram	Mean	m=0.90988	b=-0.0019788	strong
LHL	Histogram	First quartile	m=0.84025	b=-0.011086	strong
LHL	Histogram	Median	m=1.0889	b=0.0051367	strong
LLH	Histogram	Root mean square	m=1.0572	b=-0.0060701	strong

LLH	Histogram	Variance	m=1.296	b=-0.0099193	strong
LLH	Histogram	Energy	m=1.5366	b=-68.2035	strong
LLH	GLCM	Contrast	m=1.2871	b=-1.6771	strong
LLH	GLCM	Dissimilarity	m=1.0956	b=-0.11026	strong
LLH	GLCM	Informational measure of correlation 1 (IMC1)	m=1.0896	b=0.036793	strong
LLH	GLCM	Inverse Difference Moment Normalized (IDMN)	m=1.2586	b=-0.25723	strong
LLH	GLCM	Inverse Difference Normalized (IDN)	m=1.057	b=-0.055888	strong
LLH	GLCM	Inverse variance	m=1.017	b=-0.0091163	strong
LLH	GLRLM	Long Run Emphasis (LRE)	m=1.0958	b=10.234	strong
LLH	GLRLM	Run Length Nonuniformity (RLN)	m=1.2139	b=-207.1562	strong
LLH	GLRLM	Long Run High Gray-Level Emphasis (LRHGLE)	m=0.77455	b=111.8703	strong
LLH	Histogram	Mean	m=0.99997	b=-0.0030661	strong
LLH	Histogram	First quartile	m=1.0206	b=0.00049289	strong
LLH	Histogram	Median	m=1.0423	b=0.0031652	strong
LLL	Histogram	Root mean square	m=1.0564	b=-0.22442	strong
LLL	Histogram	Standard deviation	m=1.2201	b=-0.12987	strong
LLL	Histogram	Uniformity	m=1.0354	b=-0.0077893	strong
LLL	Histogram	Variance	m=1.3461	b=-0.087023	strong
LLL	Histogram	Energy	m=1.3587	b=-39999.2416	strong
LLL	GLCM	Contrast	m=1.0855	b=-1.2725	strong
LLL	GLCM	Correlation	m=1.073	b=-0.052559	strong
LLL	GLCM	Difference entropy	m=0.99392	b=-0.0048896	strong
LLL	Histogram	Entropy	m=1.1289	b=-0.19933	strong
LLL	GLCM	Dissimilarity	m=1.0261	b=-0.091335	strong
LLL	GLCM	Homogeneity 1	m=0.9473	b=0.021714	strong
LLL	GLCM	Homogeneity 2	m=0.93351	b=0.021739	strong
LLL	GLCM	Informational measure of correlation 1 (IMC1)	m=1.4358	b=0.081347	strong
LLL	GLCM	Inverse Difference Moment Normalized (IDMN)	m=1.0706	b=-0.06959	strong
LLL	GLCM	Inverse Difference Normalized (IDN)	m=1.0106	b=-0.0092556	strong
LLL	GLCM	Inverse variance	m=0.93615	b=0.022172	strong
LLL	GLRLM	Short Run Emphasis (SRE)	m=0.93602	b=0.059231	strong
LLL	GLRLM	Long Run Emphasis (LRE)	m=0.91495	b=0.13222	strong

LLL	GLRLM	Long Run Emphasis (LRE)	m=1.0658	b=2.7072	strong
LLL	GLRLM	Run Length Nonuniformity (RLN)	m=1.1199	b=-34.1273	strong
LLL	GLRLM	Run Percentage (RP)	m=0.92702	b=0.06479	strong
LLL	Histogram	Maximum	m=1.1604	b=-1.247	strong
LLL	Histogram	Mean	m=1.0468	b=-0.1745	strong
LLL	Histogram	Mean absolute deviation	m=1.211	b=-0.092567	strong
LLL	Histogram	First quartile	m=1.0022	b=0.038482	strong
LLL	Histogram	Third quartile	m=1.0569	b=-0.21749	strong
LLL	Histogram	Median	m=1.022	b=-0.028474	strong
LLL	Histogram	Minimum	m=1.032	b=-0.16221	strong
LLL	Histogram	Range	m=1.215	b=-0.82061	strong
None	GLCM	Sum Average	m=0.84835	b=5.9044	Moderate
None	GLCM	Sum entropy	m=1.13	b=-0.65971	Moderate
None	GLCM	Variance	m=0.93118	b=3.3601	Moderate
None	GLRLM	Short Run High Gray-Level Emphasis (SRHGLE)	m=0.81696	b=56.9359	Moderate
HHH	GLCM	Cluster Prominence	m=1.072	b=-1441.5731	Moderate
HHH	GLCM	Cluster Shade	m=1.072	b=-1441.5731	Moderate
HHH	GLCM	Cluster Tendency	m=0.69838	b=3.4009	Moderate
HHH	GLCM	Contrast	m=1.1911	b=-1.9589	Moderate
HHH	GLCM	Inverse Difference Moment Normalized (IDMN)	m=1.0912	b=-0.090216	Moderate
HHH	GLCM	Sum variance	m=0.69838	b=3.4009	Moderate
HHH	GLCM	Variance	m=0.79693	b=0.56632	Moderate
HHH	GLRLM	Low Gray-Level Run Emphasis (LGLRE)	m=2.2759	b=-0.0053348	Moderate
HHH	GLRLM	Short Run Low Gray-Level Emphasis (SRLGLE)	m=2.2131	b=-0.0046664	Moderate
HHH	Histogram	Mean absolute deviation	m=1.0889	b=7.0816e-05	Moderate
HHH	GLCM	Third quartile	m=1.0278	b=3.7278e-05	Moderate
HHL	GLCM	Cluster Tendency	m=0.79484	b=3.4594	Moderate
HHL	GLCM	Sum variance	m=0.79484	b=3.4594	Moderate
HLH	Histogram	Variance	m=2.0257	b=-5.0796e-05	Moderate
HLH	GLCM	Contrast	m=1.3021	b=-2.6103	Moderate
HLH	Histogram	Minimum	m=1.2039	b=-0.0040663	Moderate
HLL	GLCM	Contrast	m=2.1658	b=-8.167	Moderate
HLL	GLCM	Inverse Difference Moment Normalized (IDMN)	m=1.7495	b=-0.74524	Moderate
HLL	GLRLM	Long Run Low Gray-Level	m=1.6604	b=-0.0057476	Moderate

		Emphasis (LRLGLE)			
LHH	GLCM	Autocorrelation	m=1.0249	b=25.4796	Moderate
LHH	GLCM	Homogeneity 1	m=0.86576	b=0.052965	Moderate
LHH	GLCM	Homogeneity 2	m=0.83322	b=0.054322	Moderate
LHH	GLRLM	Short Run Emphasis (SRE)	m=0.80367	b=0.17783	Moderate
LHH	GLRLM	Long Run Emphasis (LRE)	m=0.78401	b=0.37721	Moderate
LHH	GLRLM	Short Run High Gray-Level Emphasis (SRHGLE)	m=1.0733	b=14.6552	Moderate
LHL	Histogram	Variance	m=0.40945	b=0.0026411	Moderate
LHL	GLCM	Energy	m=0.51484	b=0.0059592	Moderate
LHL	GLCM	Informational measure of correlation 2 (IMC2)	m=0.73617	b=0.22331	Moderate
LHL	GLRLM	Short Run Low Gray-Level Emphasis (SRLGLE)	m=0.71685	b=0.0017199	Moderate
LLH	GLCM	Correlation	m=1.1635	b=-0.16002	Moderate
LLH	GLCM	Sum entropy	m=0.64732	b=1.7908	Moderate
LLL	GLCM	Cluster Tendency	m=0.98021	b=7.2613	Moderate
LLL	GLCM	Sum variance	m=0.98021	b=7.2613	Moderate
LLL	GLRLM	Long Run Low Gray-Level Emphasis (LRLGLE)	m=0.89588	b=0.0010179	Moderate
HHL	Histogram	Energy	m=2.0707	b=-0.023434	Moderate
HLH	Histogram	Energy	m=2.1132	b=-0.094791	Moderate
LHH	Histogram	Energy	m=2.0924	b=-0.24824	Moderate
None	Geometric	Compactness 1	m=0.71137	b=0.0071792	Moderate
None	Geometric	Compactness 2	m=0.71503	b=0.060861	Moderate
None	Geometric	Maximum 3D diameter	m=0.84147	b=10.6605	Moderate
None	Geometric	Spherical disproportion	m=0.64702	b=0.57112	Moderate
None	Geometric	Sphericity	m=0.70473	b=0.17903	Moderate
None	GLCM	Energy	m=0.82771	b=0.001164	Moderate
None	GLCM	Entropy	m=1.009	b=-0.080026	Moderate
None	GLCM	Informational measure of correlation 2 (IMC2)	m=1.0538	b=-0.046663	Moderate
None	Histogram	Kurtosis	m=0.73172	b=-0.14234	Moderate
None	GLRLM	Long Run Low Gray-Level Emphasis (LRLGLE)	m=0.90849	b=0.00074211	Moderate
None	GLRLM	Long Run High Gray-Level Emphasis (LRHGLE)	m=0.87354	b=73.7655	Moderate
HHH	GLCM	Informational measure of correlation 2 (IMC2)	m=1.0173	b=-0.068867	Moderate
HHH	Histogram	First quartile	m=0.79783	b=-0.00012468	Moderate

HHL	Histogram	Root mean square	m=0.72361	b=0.0011964	Moderate
HHL	Histogram	Standard deviation	m=0.71009	b=0.0011911	Moderate
HHL	Histogram	Cluster Prominence	m=0.99505	b=-1177.4201	Moderate
HHL	GLCM	Cluster Shade	m=0.99505	b=-1177.4201	Moderate
HHL	GLCM	Difference entropy	m=0.76368	b=0.69238	Moderate
HHL	GLCM	Dissimilarity	m=1.0227	b=0.022608	Moderate
HHL	GLCM	Homogeneity 1	m=0.70264	b=0.13598	Moderate
HHL	GLCM	Homogeneity 2	m=0.66695	b=0.1302	Moderate
HHL	GLCM	Informational measure of correlation 2 (IMC2)	m=1.0393	b=-0.078437	Moderate
HHL	GLCM	Inverse Difference Normalized (IDN)	m=0.94095	b=0.053722	Moderate
HHL	GLCM	Inverse variance	m=0.66527	b=0.11462	Moderate
HHL	GLCM	Variance	m=0.95759	b=-0.44963	Moderate
HHL	GLRLM	Short Run Emphasis (SRE)	m=0.66981	b=0.28988	Moderate
HHL	GLRLM	Run Percentage (RP)	m=0.69132	b=0.25631	Moderate
HHL	GLRLM	Short Run Low Gray-Level Emphasis (SRLGLE)	m=0.63422	b=0.002371	Moderate
HHL	Histogram	Mean absolute deviation	m=0.72196	b=0.00078952	Moderate
HHL	Histogram	Minimum	m=1.5113	b=0.00318	Moderate
HLH	Histogram	Root mean square	m=1.4189	b=-0.002102	Moderate
HLH	Histogram	Standard deviation	m=1.4043	b=-0.0018919	Moderate
HLH	Histogram	Uniformity	m=0.91815	b=0.01355	Moderate
HLH	GLCM	Cluster Prominence	m=0.78485	b=-1633.5169	Moderate
HLH	GLCM	Cluster Shade	m=0.78485	b=-1633.5169	Moderate
HLH	GLCM	Cluster Tendency	m=0.79054	b=-1.481	Moderate
HLH	GLCM	Difference entropy	m=0.8383	b=0.44374	Moderate
HLH	Histogram	Entropy	m=0.83982	b=0.54499	Moderate
HLH	GLCM	Dissimilarity	m=0.98169	b=0.041344	Moderate
HLH	GLCM	Energy	m=0.8104	b=0.0054747	Moderate
HLH	GLCM	Entropy	m=0.77123	b=1.3602	Moderate
HLH	GLCM	Homogeneity 1	m=0.81639	b=0.088045	Moderate
HLH	GLCM	Homogeneity 2	m=0.80238	b=0.081577	Moderate
HLH	GLCM	Inverse Difference Moment Normalized (IDMN)	m=1.1973	b=-0.19567	Moderate
HLH	GLCM	Inverse Difference Normalized (IDN)	m=0.92602	b=0.069623	Moderate
HLH	GLCM	Inverse variance	m=0.78103	b=0.0782	Moderate
HLH	Histogram	Kurtosis	m=0.88218	b=0.99808	Moderate
HLH	GLCM	Inverse variance	m=0.94335	b=0.011658	Moderate

HLH	GLCM	Sum entropy	m=0.83696	b=0.57897	Moderate
HLH	GLCM	Sum variance	m=0.79054	b=-1.481	Moderate
HLH	GLCM	Variance	m=0.88417	b=-1.2714	Moderate
HLH	GLRLM	Short Run Emphasis (SRE)	m=0.82272	b=0.1536	Moderate
HLH	GLRLM	Long Run Emphasis (LRE)	m=0.78585	b=0.45413	Moderate
HLH	GLRLM	Run Percentage (RP)	m=0.84513	b=0.12534	Moderate
HLH	Histogram	Maximum	m=1.5746	b=-0.0061247	Moderate
HLH	Histogram	First quartile	m=1.1566	b=0.00017507	Moderate
HLH	Histogram	Range	m=1.4855	b=-0.0076024	Moderate
HLL	Histogram	Variance	m=0.98513	b=0.00057859	Moderate
HLL	GLCM	Cluster Prominence	m=1.2881	b=-7793.0379	Moderate
HLL	GLCM	Cluster Shade	m=1.2881	b=-7793.0379	Moderate
HLL	GLCM	Cluster Tendency	m=1.1287	b=-13.8947	Moderate
HLL	GLCM	Difference entropy	m=0.97735	b=0.1771	Moderate
HLL	GLCM	Dissimilarity	m=1.2505	b=-0.29957	Moderate
HLL	GLCM	Homogeneity 2	m=0.85932	b=0.045389	Moderate
HLL	GLCM	Informational measure of correlation 2 (IMC2)	m=1.1964	b=-0.20906	Moderate
HLL	GLCM	Inverse variance	m=0.82106	b=0.04549	Moderate
HLL	GLCM	Sum variance	m=1.1287	b=-13.8947	Moderate
HLL	GLCM	Variance	m=1.3702	b=-7.6208	Moderate
HLL	GLRLM	Short Run Emphasis (SRE)	m=0.95379	b=0.040976	Moderate
HLL	GLRLM	Long Run Emphasis (LRE)	m=1.0058	b=0.064405	Moderate
HLL	GLRLM	Run Percentage (RP)	m=0.92356	b=0.064118	Moderate
HLL	GLRLM	Long Run High Gray-Level Emphasis (LRHGLE)	m=0.77879	b=199.7316	Moderate
HLL	Histogram	Minimum	m=1.1082	b=-0.008778	Moderate
HLL	Histogram	Range	m=1.2954	b=-0.05527	Moderate
LHH	Histogram	Root mean square	m=0.74966	b=0.0037429	Moderate
LHH	Histogram	Standard deviation	m=0.74494	b=0.0037296	Moderate
LHH	GLCM	Cluster Prominence	m=1.1496	b=-3796.7446	Moderate
LHH	GLCM	Cluster Shade	m=1.1496	b=-3796.7446	Moderate
LHH	GLCM	Cluster Tendency	m=1.0684	b=-9.2888	Moderate
LHH	GLCM	Contrast	m=1.416	b=-3.8555	Moderate
LHH	GLCM	Difference entropy	m=0.69232	b=0.84121	Moderate
LHH	GLCM	Dissimilarity	m=1.1603	b=-0.29619	Moderate
LHH	GLCM	Informational measure of correlation 2 (IMC2)	m=0.9752	b=-0.02059	Moderate
LHH	GLCM	Inverse Difference Moment Normalized (IDMN)	m=1.3645	b=-0.36142	Moderate

LHH	GLCM	Inverse Difference Normalized (IDN)	m=1.1032	b=-0.098496	Moderate
LHH	GLCM	Inverse variance	m=0.9567	b=0.0079207	Moderate
LHH	GLCM	Sum Average	m=0.92294	b=4.2864	Moderate
LHH	GLCM	Sum variance	m=1.0684	b=-9.2888	Moderate
LHH	GLCM	Variance	m=1.148	b=-3.623	Moderate
LHH	GLRLM	Run Percentage (RP)	m=0.81269	b=0.16242	Moderate
LHH	GLRLM	Long Run Low Gray-Level Emphasis (LRLGLE)	m=0.58399	b=0.0030436	Moderate
LHH	Histogram	Mean absolute deviation	m=0.7536	b=0.0027097	Moderate
LHH	Histogram	First quartile	m=0.80593	b=-0.00139	Moderate
LHL	Histogram	Standard deviation	m=0.65397	b=0.021076	Moderate
LHL	GLCM	Cluster Prominence	m=0.70656	b=2848.469	Moderate
LHL	GLCM	Cluster Shade	m=0.70656	b=2848.469	Moderate
LHL	GLCM	Cluster Tendency	m=0.73421	b=14.526	Moderate
LHL	GLCM	Contrast	m=0.69002	b=4.0477	Moderate
LHL	GLCM	Difference entropy	m=0.64007	b=0.98512	Moderate
LHL	GLCM	Dissimilarity	m=0.72082	b=0.69126	Moderate
LHL	GLCM	Homogeneity 1	m=0.67959	b=0.14453	Moderate
LHL	GLCM	Homogeneity 2	m=0.67086	b=0.12601	Moderate
LHL	GLCM	Inverse Difference Moment Normalized (IDMN)	m=0.70565	b=0.29083	Moderate
LHL	GLCM	Inverse Difference Normalized (IDN)	m=0.72149	b=0.25981	Moderate
LHL	GLCM	Inverse variance	m=0.71777	b=0.099513	Moderate
LHL	GLCM	Sum variance	m=0.73421	b=14.526	Moderate
LHL	GLCM	Variance	m=0.74859	b=4.1394	Moderate
LHL	GLRLM	Short Run Emphasis (SRE)	m=0.66144	b=0.30021	Moderate
LHL	GLRLM	Long Run Emphasis (LRE)	m=0.64207	b=0.64885	Moderate
LHL	GLRLM	Run Percentage (RP)	m=0.6849	b=0.26584	Moderate
LHL	Histogram	Maximum	m=0.88462	b=0.020972	Moderate
LHL	Histogram	Mean absolute deviation	m=0.67643	b=0.015334	Moderate
LHL	Histogram	Third quartile	m=1.1071	b=0.0023946	Moderate
LHL	Histogram	Minimum	m=0.77128	b=-0.067997	Moderate
LHL	Histogram	Range	m=0.86806	b=0.06089	Moderate
LLH	Histogram	Standard deviation	m=1.0826	b=-0.011418	Moderate
LLH	GLCM	Autocorrelation	m=0.87433	b=40.0353	Moderate
LLH	GLCM	Cluster Prominence	m=0.61516	b=9025.6656	Moderate
LLH	GLCM	Cluster Shade	m=0.61516	b=9025.6656	Moderate
LLH	GLCM	Cluster Tendency	m=0.65616	b=26.7418	Moderate

LLH	GLCM	Difference entropy	m=0.97071	b=0.13536	Moderate
LLH	GLCM	Energy	m=0.59879	b=0.0035909	Moderate
LLH	GLCM	Entropy	m=0.74344	b=1.9087	Moderate
LLH	GLCM	Homogeneity 1	m=0.86135	b=0.054727	Moderate
LLH	GLCM	Homogeneity 2	m=0.84288	b=0.051211	Moderate
LLH	GLCM	Informational measure of correlation 2 (IMC2)	m=0.84902	b=0.12889	Moderate
LLH	GLCM	Sum Average	m=0.87976	b=4.2325	Moderate
LLH	GLCM	Sum variance	m=0.65616	b=26.7418	Moderate
LLH	GLCM	Variance	m=0.73312	b=5.8139	Moderate
LLH	GLRLM	Short Run Emphasis (SRE)	m=0.75097	b=0.22574	Moderate
LLH	GLRLM	Long Run Emphasis (LRE)	m=0.66362	b=0.54016	Moderate
LLH	GLRLM	Run Percentage (RP)	m=0.75978	b=0.20942	Moderate
LLH	Histogram	Maximum	m=0.86384	b=0.085699	Moderate
LLH	GLRLM	Short Run Low Gray-Level Emphasis (SRLGLE)	m=0.76808	b=0.0014878	Moderate
LLH	GLRLM	Short Run High Gray-Level Emphasis (SRHGLE)	m=0.78436	b=65.9727	Moderate
LLH	Histogram	Mean absolute deviation	m=1.1244	b=-0.016822	Moderate
LLH	Histogram	Third quartile	m=1.2707	b=-0.0075729	Moderate
LLH	Histogram	Minimum	m=1.0907	b=0.03045	Moderate
LLH	Histogram	Range	m=1.022	b=0.031265	Moderate
LLL	GLCM	Autocorrelation	m=0.8966	b=50.3396	Moderate
LLL	GLCM	Energy	m=0.84078	b=0.0010271	Moderate
LLL	GLCM	Entropy	m=1.0457	b=-0.37011	Moderate
LLL	GLCM	Informational measure of correlation 2 (IMC2)	m=1.086	b=-0.075232	Moderate
LLL	Histogram	Kurtosis	m=0.68943	b=-0.16314	Moderate
LLL	GLCM	Sum Average	m=0.89333	b=4.6496	Moderate
LLL	GLCM	Sum entropy	m=1.0977	b=-0.49335	Moderate
LLL	GLCM	Variance	m=0.93225	b=3.3225	Moderate
LLL	GLRLM	Short Run Low Gray-Level Emphasis (SRLGLE)	m=0.98879	b=-0.00037913	Moderate
LLL	GLRLM	Short Run High Gray-Level Emphasis (SRHGLE)	m=0.89859	b=41.7885	Moderate
LLL	GLRLM	Long Run High Gray-Level Emphasis (LRHGLE)	m=0.90641	b=66.0855	Moderate
